# Real-world evaluation of safety and efficacy of AHCL systems in young children with type 1 diabetes: a 1-year assessment

**DOI:** 10.3389/fendo.2025.1590964

**Published:** 2025-06-26

**Authors:** Daniele Franzone, Giordano Spacco, Andrea Piano, Giulia Siri, Giacomo Tantari, Giuseppe d’Annunzio, Maria Grazia Calevo, Mohamad Maghnie, Nicola Minuto, Marta Bassi

**Affiliations:** ^1^ DINOGMI (Department of Neuroscience, Rehabilitation, Ophthalmology, Genetics, Maternal and Child Health), University of Genoa, Genoa, Italy; ^2^ Department of Pediatrics and Neonatology, Istituto di Ricovero e Cura a Carattere Scientifico (IRCCS) Istituto Giannina Gaslini, Savona, Italy; ^3^ Pediatric Clinic, Istituto di Ricovero e Cura a Carattere Scientifico (IRCCS) Istituto Giannina Gaslini, Genoa, Italy; ^4^ Biostatistics Unit, Scientific Directorate, Istituto di Ricovero e Cura a Carattere Scientifico (IRCCS) Istituto Giannina Gaslini, Genoa, Italy

**Keywords:** type 1 diabetes, toddlers, AID (automated insulin delivery), AHCL (advanced hybrid closed loop), off-label

## Abstract

**Background and aims:**

Management of Type 1 Diabetes (T1D) in young children is challenging. A poor glycaemic control during the first years of disease increases the risk of microvascular complications. Moreover, hyperglycaemia and glucose variability have a negative effect on the brain development. Advanced hybrid closed loop (AHCL) systems demonstrated to improve glycaemic control in adolescents and adults with T1D although data on younger children are limited. The aim of the study was to evaluate the safety and the effectiveness of AHCL systems’ off-label use in children aged less than 7 years.

**Methods:**

A retrospective single-center study on T1D patients aged less than 7 years using AHCL systems was conducted. Glycated hemoglobin (HbA1c) values, Continuous Glucose Monitoring (CGM) and insulin requirement data were collected at T0 (AHCL starting), T1 (1-month), T2 (3-months) and T3 (1-year).

**Results:**

41 patients were included in the study. No episode of severe hypoglycaemia occurred. Three patients experienced an episode of ketoacidosis (DKA) due to insulin delivery set occlusion. During the 12-months study period, an improvement in HbA1c value (7.50 *vs* 6.59%, p<0.001), Time in Range (TIR, +10.21%, p<0.001) and Time in Tight Range (TITR, +7.56%, p=0.003) were observed, with a reduction in time in hyperglycaemia and without an increase in time in hypoglycaemia. The AHCL use increased insulin requirement at 12-months, especially in bolus doses (p<0.001).

**Conclusions:**

Although AHCL systems are not currently approved for this age group, we have demonstrated their safety and efficacy in children under 7 years with T1D. The use of these systems resulted in significant improvement in glycaemic control without increasing the risk of hypoglycaemia. The impact of early glycaemic control on brain development during the first years of life may support the early introduction of AHCL systems in very young children with T1D. It is essential to gather data that could support the approval of these systems for use in younger age groups.

## 1 Introduction

Management of Type 1 Diabetes (T1D) in young children is challenging. Most preschoolers do not reach recommended glycaemic targets due to high glycaemic variability and unpredictable daily exercise activities and meals (1, 2). The fear of hypoglycaemia is common in caregivers, leading to an overwhelming burden of care for parents with an impact on glycaemic control, sleep and overall well-being (3, 4). Hyperglycaemia and high glycaemic variability during the first years of life increase the risk of microvascular complications and have a negative effect on the brain development and cognitive and executive functions (5). Consequently, an intensive management of T1D and a tight glycaemic control are recommended in younger children (6).

The huge technological advancements achieved during the past decade, in particular the introduction of Automated Insulin Delivery (AID) systems, created a paradigm shift in T1D standards of care helping to improve glycaemic control and psychological well-being of patients (7, 8).

Currently, two AID systems are approved for use in very young children: CamAPS FX (CamDiab^®^, Cambridge, UK), approved from 1 year of age, and Omnipod 5 (Insulet^®^, Acton, MA, USA), approved from 2 years of age (9–11). These devices are commonly referred to as Hybrid Closed Loop (HCL) systems, as they modulate basal insulin delivery but do not deliver automatic correction boluses. Tandem Control-IQ (Tandem Diabetes Care^®^, San Diego, CA) and MiniMed 780G (Medtronic^®^, Northridge, CA) incorporate this feature and are therefore classified as Advanced Hybrid Closed Loop (AHCL) systems. Both have shown comparable results in terms of safety and efficacy in pediatric populations (12, 13). Tandem Control-IQ is an AHCL system approved for use from 6 years of age and a minimum daily insulin dose of 10 U/day in both USA and Europe. The integrated Model Predictive Control (MPC) algorithm is able to adjust basal insulin delivery and to deliver a correction bolus in case of hyperglycaemia every hour. Approval and real world studies, both in children 6–13 years of age and in adults and adolescent > 14 years confirmed safety and efficacy of Tandem Control-IQ (14–16). To date, only a few studies demonstrated safety and efficacy of this system in very young children (17–20).

MiniMed 780G (Medtronic^®^, Northridge, CA) is an AHCL system empowered with an interoperable Predictive Integrative Derivative (PID) algorithm, approved for use from 7 years of age. The SmartGuard^®^ automatic mode works for daily insulin dosages above 8 U/day and is able to adapt basal insulin delivery and to deliver a correction bolus in case of hyperglycaemia every 5 minutes. Pivotal trials and real-world studies in adolescents and adults with T1D established the safety and the greater effectiveness of the system compared to previous standards of care in terms of glycaemic outcomes and patients’ satisfaction (21–23). Safety and effectiveness of the system in very young children was demonstrated as well along with a reduction in diabetes related parental stress (20, 24–28).

The aim of this study was to evaluate the safety and effectiveness of the off-label use of AHCL systems in preschoolers in a 1 year real-world period.

## 2 Materials and methods

A retrospective single center study was conducted at the Regional Pediatric Diabetes Center of IRCCS Giannina Gaslini Institute (Genoa, Italy) to evaluate the safety and the effectiveness of the off-label use of two AHCL systems in very young children. The safety was evaluated through the assessment of number of episodes of severe hypoglycaemia (SH) or diabetic ketoacidosis (DKA) in the first 12-months of use. The effectiveness was evaluated in terms of improvement in Time in Range (TIR) and other glucose sensor metrics after 1-year of use.

A total of 41 pediatric patients were included, considering the following inclusion criteria:

- diagnosis of T1DM according to ISPAD guidelines (29)
- age at start of AHCL therapy < 7 years for Minimed 780G and < 6 years for Tandem Control-IQ
- use of an AHCL system for at least 1 year
- Total Daily Insulin Dose (TDI) > 8 UI, for the activation of SmartGuard, or > 10 UI, for the activation of Control-IQ algorithm.

Exclusion criteria were: conditions or medications affecting glycaemic levels, hemophilia or any other bleeding disorders and algorithm utilization time less than 90%. All parents or caregivers provided written informed consent to the off-label use of the algorithm-driven automated insulin deliver. Ethics committee approval was not requested, since the General Authorization to Process Personal Data for Scientific Research Purposes (authorization no. 9/2014) declared that retrospective archive studies that use identifier codes, preventing the data from being traced back directly to the data subject, do not need ethics approval.

All patients included in the study initiated AHCL therapy during a dedicated in-hospital session. A standardized 3-hour training was delivered by the diabetes team, including education on pump functioning, setting of initial parameters, first infusion set placement under supervision of a trained nurse and demonstration of the pump main features (i.e. bolus delivery, exercise mode). If needed, a 30-minute carbohydrate-counting session was conducted by a specialized dietitian. For MiniMed 780G, the transition from manual to automatic mode typically occurred within 3 to 5 days.

**System settings**: For Tandem Control-IQ, glycemic target and active insulin time (AIT) are fixed and not user-adjustable; therefore, uniform settings were applied to all patients using this system.

For Minimed 780G, glycemic target and active insulin time (AIT) are customizable settings. According to manufacturer recommendations, the optimal configuration includes a glycemic target of 100 mg/dL and an AIT of 2 hours. However, in our cohort of very young children treated off-label, more conservative settings have been used in several cases to reduce the risk of hypoglycemia. These individual settings were not systematically collected and varied across patients, but most of them were using a glycemic target of 100 mg/dl and an active insulin time of 3 hours during the observational period.

Demographical and clinical data were collected from electronic clinical records of regular follow-up visits at T0 (starting of AHCL system in automatic mode, Smartguard^®^ or Control-IQ^®^) and after 1-month (T1), 3-months (T2) and 12-months (T3) after starting algorithm-driven insulin delivery.

Age, gender, weight, age at T1D onset, duration of disease, previous insulin therapy, total daily insulin dose (TDI, U/die) and glycated hemoglobin (HbA1c) were collected at T0.

CGM data of the previous 14-day period were collected at T0, T1, T2 and T3 and included: Time in Range (TIR, 70–180 mg/dl), Time in Tight Range (TITR, 70–140 mg/dl), Time Above Range (TAR, 180–250 mg/dl), TAR>250 (>250 mg/dl), Time Below Range (TBR, 54–70 mg/dl), TBR<54 (<54 mg/dl), Glucose management indicator (GMI%), Average Glucose (AG, mg/dl) value, Standard Deviation (SD, mg/dl), glucose coefficient of variation (CV%) and percentage of sensor use. CGM and insulin pump data were collected remotely through data sharing dedicated platforms.

Furthermore, in order to evaluate changes in insulin requirements with AHCL systems, data were analyzed for insulin need at different times, not only in terms of total dosage but also in terms of bolus/basal ratio. Weight, total daily insulin dose (TDI, U/kg/die), bolus daily dose (U/kg/die) and basal daily dose per kg (U/kg/die) were collected at all the study times.

Number of episodes of severe hypoglycaemia (SH) or diabetic ketoacidosis (DKA) and skin reactions were reported for all the 12-months study period.

### 2.1 Statistical analysis

A descriptive analysis has been performed. Data are described as mean and standard deviation (SD) or median and range for continuous variables, and as absolute and relative frequencies for categorical variables. The Kolmogorov–Smirnov statistical test was used to assess the normal data distribution. Comparisons between T0, T1, T2 and T3 to examine continuous nonparametric variables were performed using Paired Wilcoxon test. P values ≤ 0.05 were considered statistically significant, and all P values were based on two tailed tests. Statistical analysis was performed using SPSS Statistical Package for the Social Sciences for Windows version 29 (SPSS Inc. Chicago, IL USA).

## 3 Results

Population characteristics at baseline (T0) are summarized in **Table 1**. 41 pediatric patients (26 boys; 15 girls) with T1D (with a median age at disease onset of 2.80 ± 1,43) and using AHCL systems (23 MiniMed 780G; 18 Tandem Control-IQ) were included in the study. The median age at T0 was 4.41 ± 1.61 and the median duration of disease was 1.61 ± 1.44 years.

**Table 1:** Population characteristics at baseline (T0).

	Mean, median or frequency (total n=41)
Age (years)	4,41 ± 1,61
Female	15 (36,6%)
Weight (kg)	18,44 ± 4,96
Age at T1D diagnosis (years)	2,80 ± 1,43
Duration of T1D at T0 (years)	1,61 ± 1,44
HbA1c (%)	7.50 ± 1.14
Type of AHCL system	8,64 ± 1,81
Tandem Control IQ	18 (43,9%)
Minimed 780G	23 (56,1%)
Previous Therapy	
PLGS	28 (68,3%)
MDI	9 (22%)
No Therapy	4 (9,7%)

Data are described as mean and standard deviation (SD) or median and range for continuous variables, and as absolute and relative frequencies for categorical variables.

**Table 2** reports CGM metrics, HbA1c values, insulin requirement and sensor use expressed as median values and standard deviations (SD) at baseline, 1-month, 3-months, and 1-year after transition to AHCL algorithm-driven insulin delivery.

**Table 2:** CGM metrics, HbA1c, insulin requirement and % sensor use at T0, T1 (1 month), T2 (3 months) and T3 (1 year) after initiation of AHCL system.

	T0	T1	P (T1vsT0)	T2	P (T2vsT0)	T3	P (T3vsT0)
TIR% (70–180 mg/dl)	55,50 ± 13,20	67,09 ± 8,70	**<0,001**	66,18 ± 7,61	**<0,001**	65,71 ± 9,14	**<0,001**
TTIR% (70–140 mg/dl)	36,00 ± 10,42	45,36 ± 8,29	**<0,001**	45,04 ± 8,04	**<0,001**	43,56 ± 9,33	**0,003**
TAR% (181–250 mg/dl)	24,26 ± 7,25	20,00 ± 4,59	**<0,001**	20,06 ± 5,90	**0,002**	20,91 ± 5,74	**0,01**
TAR% (>250 mg/dl)	16,06 ± 11,55	9,03 ± 5,99	**<0,001**	9,76 ± 5,62	**0,003**	9,43 ± 5,59	**0,002**
TBR % (55–69 mg/dl)	3,18 ± 2,36	3,32 ± 1,95	0,73	3,18 ± 2,24	0,53	3,23 ± 2,02	0,72
TBR % (<54 mg/dl)	0,85 ± 0,99	0,88 ± 1,12	0,75	0,85 ± 1,05	0,75	0,94 ± 1,11	0,56
HbA1c %	7,50 ± 1,14	7,26 ± 0,96	**0,02**	6,68 ± 0,56	**<0,001**	6,59 ± 0,52	**<0,001**
GMI (%)	7,38 ± 0,72	6,97 ± 0,35	**<0,001**	7,01 ± 0,37	**0,002**	7,01 ± 0,37	**0,007**
SD (mg/dl)	69,20 ± 13,01	61,37 ± 10,98	**<0,001**	62,61 ± 10,15	**0,002**	61,71 ± 11,12	**0,004**
CV (%)	40,71 ± 5,62	40,53 ± 4,85	0,87	40,74 ± 4,76	0,89	39,98 ± 5,64	0,34
AG (mg/dl)	170,55 ± 30,02	152,55 ± 15,00	**<0,001**	154,59 ± 15,51	**0,002**	154,94 ± 15,33	**0,01**
TDI (u/kg/day)	0,68 ± 0,15	0,72 ± 0,14	0,07	0,76 ± 0,15	**0,01**	0,78 ± 0,19	**0,009**
Bolus (u/kg/day)	0,39 ± 0,15	0,42 ± 0,10	**0,03**	0,45 ± 0,11	**0,002**	0,47 ± 0,12	**<0,001**
Basal (u/kg/day)	0,29 ± 0,08	0,29 ± 0,08	0,51	0,30 ± 0,08	0,40	0,33 ± 0,12	0,37
Sensor use (%)	94,23 ± 5,97	94,00 ± 9,54		96,22 ± 2,74		94,98 ± 5,00	

HbA1c, Glycated Hemoglobin.

TIR, Time in Range.

TAR, Time Above Range.

TBR, Time Below Range.

AG, Average Glucose.

SD, Standard Deviation.

CV, Coefficient of Variation.

TDI, Total Daily Insulin.

(bold: statistically significant).

After starting AHCL system in automatic mode, TIR and TITR showed a statistically significant improvement at 1-month that was maintained at 3-months and 1 year (+10.21% and +7.56% respectively; from 55.50 ± 13.20 to 65.71 ± 9.14 at T3, p<0.001 for TIR and from 36.00 ± 10.42 to 43.56 ± 9.33 at T3, p=0.003 for TITR). Time spent in hyperglycaemia decreased of -3.35% for TAR (p=0.01) and -6.63% for TAR>250 (p=0.002). HbA1c% decreased all over the study period from 7.50 ± 1.14 at T0 to 6.59 ± 0.52 at 12-months (-0.91%, p<0.001) and average sensor glucose (AG) showed a significant reduction at 1-year (-15.61 mg/dl, p=0.01).

**Figure 1:**
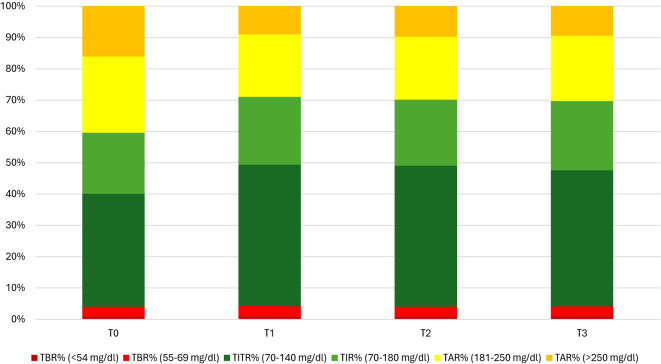
Achievement of Glycemic recommended target through the 1-year period.

**Figure 2:**
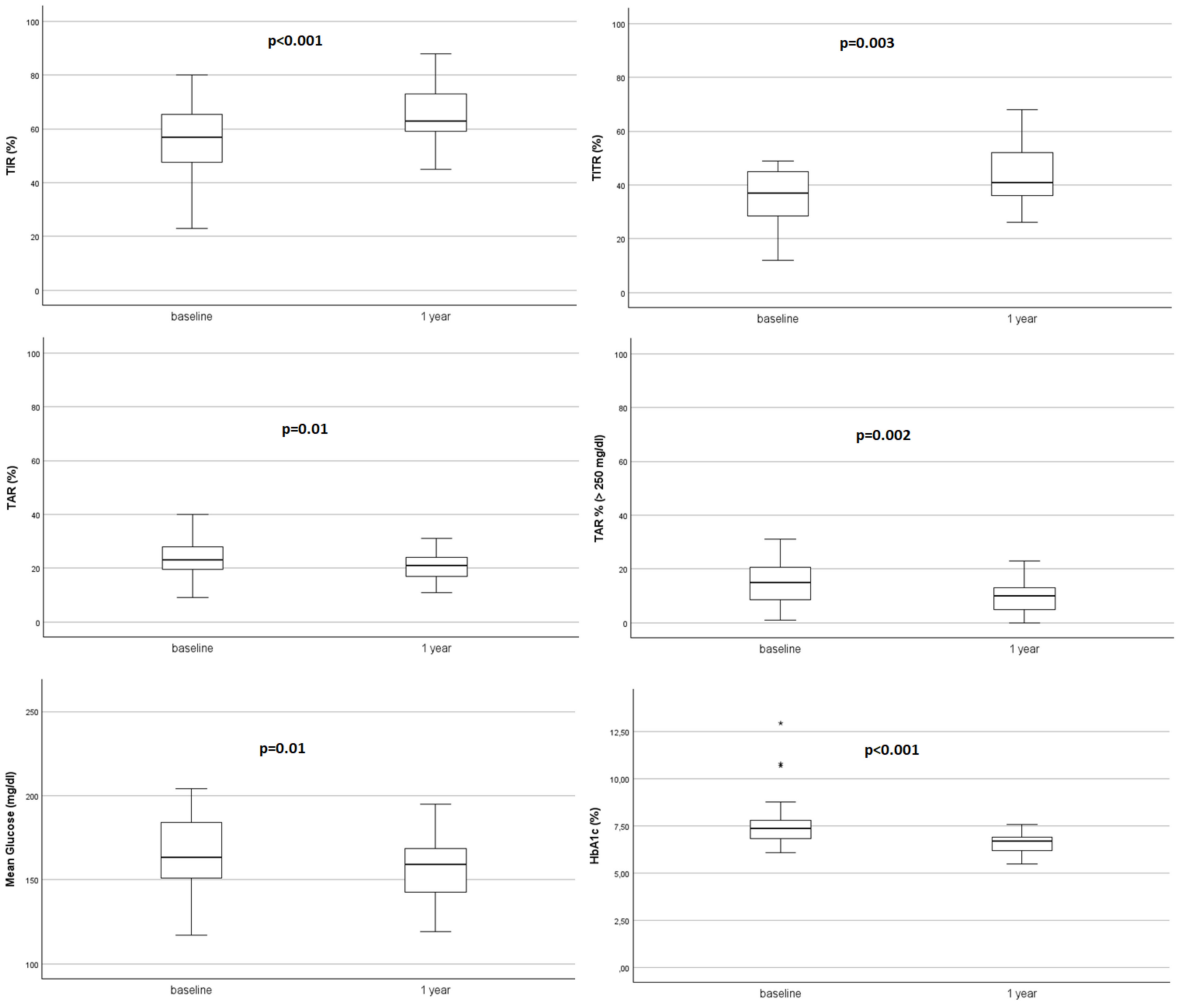
Improvement of CGM metrics through the 1-year period.

TIR and TITR improvement, as the reduction in hyperglycaemia, were obtained without an increase in time in hypoglycaemia since both TBR and TBR<54 mg/dl did not show an increase during the study period and their median values achieved recommended glycaemic targets (30). **Figures 1**, **2** show CGM metrics modifications during the 12-months follow up period.

Glycaemic variability, measured as Coefficient of variation, did not show a reduction all over the 1-year study period. Median percentage of CGM sensor use remained always satisfactory, ranging from 94% at T0 and T1 to 96% at T2 and 95% at T3. No persistent shut-downs of algorithms due to low insulin requirements were observed during the study period.

Starting the AHCL use determined an increased insulin requirement (TDI, U/kg/die) from 0.68 ± 0,15 at T0 to 0.78 ± 0.19 at T3; p=0.09), highly determined by the increase in bolus doses (U/kg/die from 0.39 ± 0.15 at T0 to 0.47 ± 0.12 at T3, p<0.001).

No episodes of SH occurred during the study period. Three patients experienced an episode of DKA due to insulin delivery set occlusion. One patient required to change AHCL system due to skin reaction to adhesive device. No other clinically relevant skin reactions were observed.

## 4 Discussion

The results of this study confirm the safety and effectiveness of AHCL systems in very young children, demonstrating improvements in glycaemic control (HbA1c and CGM metrics). While the efficacy of insulin pumps and AHCL systems is well documented in adolescents and adults with T1D, and supported by a consistent number of real-world studies, there is a limited number of studies examining their use in young children. To date, CamAPS FX and Omnipod 5 are the only algorithms approved for use in children under six years of age, while AHCL systems have not yet received approval for use in very young children (9–11).

The first application of Tandem Control-IQ in children aged 2–6 years was described in a brief pilot study involving 12 patients conducted in a home setting for 72 hours after an outpatient supervised hotel setting lasting 48 hours. In this brief pilot study, the system has been proven to be safe and improved glycaemic control (17). Moreover, a 13-week multicenter trial comparing closed loop system and standard of care in a 102 children cohort (with a 2:1 randomization ratio) demonstrated safety and effectiveness with an improvement of +12.6% in TIR during the study period (18). A recent trial demonstrated that a lower treatment range and late bolus feature of a modified Control-IQ system are safe for use in these age group (19).

Safety and impact of MiniMed 780G on glycaemic control in children aged 2–6 years has been first evaluated in a 12-week prospective study on a cohort of 35 patients. No events of SH or DKA were reported and a significant increase in TIR (+8,3%) was showed along with a reduced parental diabetes stress (24). These results were confirmed in a retrospective 9-months study conducted on 12 children which were running in automatic mode even with a TDI <8 U/day (25). Moreover, feasibility of MiniMed 780G was reported in a 6-week prospective single arm study following a 2-week manual mode phase showing the achievement of recommended CGM targets (26). Over the past years, few studies involving small patient cohorts and case reports have supported the safety and efficacy of the system in young children, and in a small group of patients with neonatal diabetes (27, 28).

More recently, a two-center prospective study, enrolling 19 children who started AHCL (MiniMed 780G or Tandem Control-IQ), switching either from MDI or open-loop insulin therapy, showed an improvement of 10% in TIR, with reduction of TAR and HbA1c after 6 months of follow-up with a small increase in TBR (20).

Given the limitation of data on the efficacy and safety of AHCL in young children and differently from previous studies, we performed a single center retrospective study among a large cohort of young patients followed up for a 12-months period, starting off-label use of AHCL, regardless of the type of system. Our results are consistent with previous literature data and confirm effectiveness of AHCL system in young children showing an improvement in TIR of 10.2% (p<0.001) and a reduction of HbA1c of 0.91% (p<0.001) at 1-year follow-up.

Unlike previous studies, we focus on Time in Tight Range (TITR), defined as the percentage of time with glucose values between 70 and 140 mg/dL. TITR is increasingly recognized as a reliable marker of good glycemic control in pediatric diabetes. This CGM-derived metric has shown potential to better capture hyperglycemia and glycemic variability, and to support individualized targets in clinical practice (31, 32). Recent real-world studies have demonstrated its clinical relevance in children and adolescents using AID systems (33, 34). In our cohort, the median TITR improved significantly over 12 months (+7.56%, *p* = 0.003), although the proposed target of ≥50% was not reached. Several factors may have contributed to this result, including the heterogeneity of the study population, the use of two different AHCL systems with distinct algorithmic behaviors, and the absence of standardized device settings across participants. Achieving such tight glycemic targets remains particularly challenging in very young children, who typically exhibit greater glucose fluctuations, irregular eating patterns, and increased insulin sensitivity (2). Nevertheless, we fully share the recent expert opinion suggesting that a 50% TITR goal may be realistic in this population when using advanced diabetes technologies (35). Our study was not designed to define the optimal strategy to reach that threshold, but rather to document the real-life experience of AHCL use in a heterogeneous and off-label pediatric setting. Future randomized controlled trials will be needed to identify the most effective strategies for achieving this goal in preschool-aged children.

**Figure 3:**
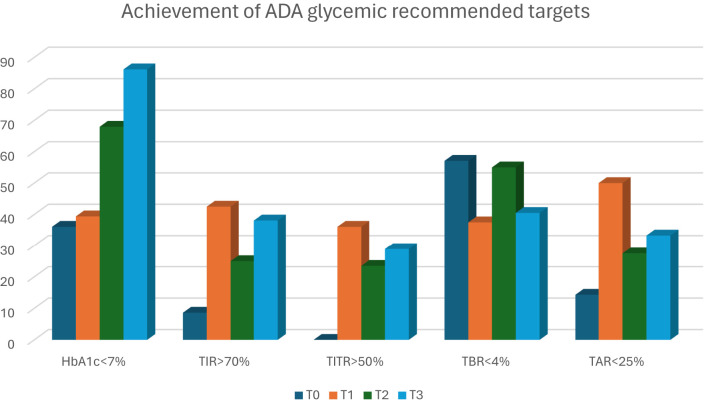
Percentage of patients achieving recommended target during the study-period.

Interestingly and consistently with the other studies, the improvement of TIR and TITR was already evident after 1-month and was maintained along the 12-months follow-up period, suggesting the prompt effect of AHCL systems on glycaemic control and confirming that a quick improvement in CGM metrics is possible even in young children. At 1 year after the initiation of AHCL, the percentage of patients achieving HbA1c values below 7% more than doubled (from 36.1% to 86.2%), the percentage of patients achieving 70% of TIR increased by 29.5% (from 8.6% to 38.1%), and the percentage of patients achieving 50% of TITR increased from 0% to 28.9% (**Figure 3**). These results show a marked improvement in achieving the glycemic targets recommended, especially considering the poor baseline control and difficulties typical of this age group. The ability to improve glycaemic control very quickly is crucial in such a critical period of life and growth in which an optimal glycaemic control is essential to guarantee an adequate cognitive development (5). The improvements in TIR and TITR have been obtained at the expense of TAR, in particular of TAR>250, with a reduction of nearly 10% (-2.4 hours/day in hyperglycaemia) at 12-months. Given the vulnerability of the brain to metabolic insults during development, T1D can have a significant impact on brain structure and cognitive functioning. Exposure to chronic hyperglycaemia and glycaemic extremes has been recognized as a risk factor for cognitive impairment, and poor glycaemic control was found to have negative effects on the memory function of children with T1D (36).

Improved glycaemic control (TIR, TITR and HbA1c) was achieved without an increased time in hypoglycaemia since TBR and TBR<54 median values met the recommended target (30) throughout the whole follow-up period and no episodes of severe hypoglycaemia were reported. As for hyperglycaemia, time spent in hypoglycaemia (in particular severe hypoglycaemia) has been associated with specific cognitive dysfunctions (37). Fear of hypoglycaemia is common among caregivers of young children with T1D and is associated to an overwhelming burden of care for parents which can impact on overall well-being. The first study focusing on the psychosocial aspects of AHCL system use in pediatric T1D patients showed the impact of these systems on caregivers’ sleep quality and capability to improve confidence in monitoring children’s therapy (38).

Regarding insulin dosage, an increase in daily insulin requirements was observed after starting AHCL (+0.1 U/kg/die, p=0.09), primarily driven by a rise in bolus insulin (p<0.001), while basal insulin remained stable. This increase may be partially explained by the functioning of the algorithms, particularly the automatic correction bolus feature. Notably, MiniMed 780G delivers automatic boluses as frequently as every 5 minutes, whereas Control-IQ applies them at most once per hour. These differences may have contributed to the variability in bolus delivery. The improvement in glycemic control observed in our cohort is likely supported, at least in part, by the automatic bolus function of AHCL systems. In both systems, minimum daily insulin requirements are specified by the manufacturers for algorithm activation (8 U/day for MiniMed 780G and 10 U/day for Tandem Control-IQ). While the MiniMed 780G system may exit automatic mode if insulin needs fall below 8 U/day, in our experience such shutdowns are rare and tend to occur only when insulin doses remain consistently below 5 U/day for prolonged periods. Tandem Control-IQ, on the other hand, does not automatically deactivate once the algorithm is running, even if total daily insulin temporarily falls below 10 U. In our cohort of patients no persistent deactivations due to low insulin requirements were observed in during the study period.

Both AHCL systems proved to be safe in young children since no episodes of severe hypoglycaemia occurred during the study period, while 3 patients reported an episode of diabetic ketoacidosis. DKA can occur in patients on insulin pump therapy as a consequence of an infusion set occlusion or failure. Education of patients and families on the correct management of the infusion set and a rapid recognition of occlusion signals (and need of replacement) is essential, especially dealing with young children, in order to avoid episodes of ketoacidosis. The safety data emerging from our study are consistent with literature (17–28). During the 12 months follow-up period few mild skin reactions have been reported. Skin reactions currently represent an emerging problem in diabetology: longer lasting CGM sensor and infusion sets are reducing burden of care for patients and families in terms of time spent for therapy and injection pain, but a prolonged contact time of patches on the skin increases the risk of skin reactions, which are even more common during summer period (39). As a consequence, a patient can decide to dismiss the insulin pump or going back to a previously used and more tolerated insulin delivery system. In our study, during the 12-months follow-up period only one patient experienced a significant skin reaction leading to a switch to another AHCL system. Given the limitation of literature data, future studies focusing on prevention and treatment of skin reactions are needed.

This study has strengths and limitations. The study demonstrated that AHCL systems are safe and effective in younger children without increasing the risk of hypoglycaemia; all patients have almost reached the glycaemic recommended target during a period of life so important for adequate brain and cognitive development: the adverse events are comparable to those reported in the literature, providing reassurance regarding the overall safety of these systems; the study offer valuable real-world data on a quite large group of patients, contributing to the understanding of how these systems work in clinical practice; the study involved different types of AHCL system and suggest that all these systems can be safely used in younger children; the study also evaluated the impact on glycaemic control in terms of TITR, a promising and recently introduced CGM metric developed to further optimize glycaemic control. The limitations are related to the absence of a comparison group of patients who used standard insulin therapy, which could limit the ability to directly compare the outcomes of AHCL systems with other approaches.

## 5 Conclusions and future perspectives

AHCL systems proved to be safe and effective in children under 7 years with T1D, although they are not yet approved for this age group. The potential long-term neurological effects of inadequate glycaemic control may justify the early introduction of AHCL system in younger children with T1D. The role of the Diabetes Team in the regular follow-up remains essential for providing continuous therapeutic education on the disease management and the correct use of technological devices, aiming to optimize glycaemic control and improve the quality of life for both the patients and their families. Collecting data to support the approval of these systems for use in younger children is crucial.

## Data Availability

The original contributions presented in the study are included in the article/supplementary material. Further inquiries can be directed to the corresponding author.
